# Experimental Methods for Studying the Contact Mechanics of Joints

**DOI:** 10.1155/2023/4914082

**Published:** 2023-09-22

**Authors:** Radovan Zdero, Pawel Brzozowski, Emil H. Schemitsch

**Affiliations:** ^1^Orthopaedic Biomechanics Lab, Victoria Hospital, London, Canada; ^2^Division of Orthopaedic Surgery, Western University, London, Canada

## Abstract

Biomechanics researchers often experimentally measure static or fluctuating dynamic contact forces, areas, and stresses at the interface of natural and artificial joints, including the shoulders, elbows, hips, and knees. This information helps explain joint contact mechanics, as well as mechanisms that may contribute to disease, damage, and degradation. Currently, the most common in vitro experimental technique involves a thin pressure-sensitive film inserted into the joint space; but, the film's finite thickness disturbs the joint's ordinary articulation. Similarly, the most common in vivo experimental technique uses video recording of 3D limb motion combined with dynamic analysis of a 3D link-segment model to calculate joint contact force, but this does not provide joint contact area or stress distribution. Moreover, many researchers may be unaware of older or newer alternative techniques that may be more suitable for their particular research application. Thus, this article surveys over 50 years of English-language scientific literature in order to (a) describe the basic working principles, advantages, and disadvantages of each technique, (b) examine the trends among the studies and methods, and (c) make recommendations for future directions. This article will hopefully inform biomechanics investigators about various in vitro and in vivo experimental methods for studying the contact mechanics of joints.

## 1. Introduction

Biomechanics researchers are interested in the static and fluctuating dynamic contact forces, areas, and stresses at the interface of natural and artificial joints, like the shoulder, elbow, wrist, hip, knee, and ankle [1]. However, aging, illness, and injury to the cartilage of natural joints can stimulate the production of enzymes that destroy the cartilage matrix and, thus, disrupt joint contact mechanics; eventually, this can lead to osteoarthritis, debilitating pain, functional loss, and the need for joint replacement surgery [2]. Similarly, a variety of wear mechanisms caused or amplified by fatigue loading of artificial joints can damage component surfaces and, thus, alter joint contact mechanics; eventually, this can lead to excessive wear debris leading to bone resorption (i.e., osteolysis), implant loosening and failure, and revision surgery [2]. As a result, obtaining accurate data on joint contact mechanics is vital, so effective strategies can be developed to reduce or eliminate mechanisms leading to joint disease, damage, and/or degradation.

**Figure 1:**
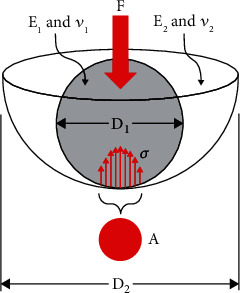
An idealized ball-and-socket joint mimicking a shoulder or hip. Diameter *D*, elastic modulus *E*, or Poisson's ratio *ν* may or may not be known. However, in vitro or in vivo experimental techniques can still be used to determine joint contact force *F*, area *A*, or stress *σ*.

The contact mechanics of 2-body interfacial articulations, like natural and artificial joints, can be partly understood from classic Hertzian theory [3, 4]. Hertzian formulas are ideally suited for quasistatic normal loads, noncongruent frictionless surfaces, and small elastic deformations at the interface, which is not always true for real joints. Even so, consider a ball-and-socket joint (e.g., shoulder or hip) involving a convex spherical ball articulating against a concave spherical socket (Figure 1). If the applied force is *F*, diameter is *D*, elastic modulus is *E*, and Poisson's ratio is *ν*, then the interface's contact area *A* will have a circular or cup shape (Equation (1)). This Hertzian formula implies that contact area *A* increases for heavier people or more intense activities (i.e., larger *F*), softer materials (i.e., smaller *E*), or better congruity (i.e., *D*_1_~*D*_2_), and vice versa. Also, by definition, a larger contact area *A* will reduce contact stress, since average contact stress *σ* = *F*/*A* and peak contact stress *σ* = 1.5*F*/*A*, and vice versa. Similar Hertzian equations and observations can be provided for other 2-body articulations relevant to joint contact mechanics involving conical, cylindrical, elliptical, and flat bodies.

(1) $$\begin{array}{c} A=\pi {\left[\frac{\left(3F/8\right)\left(\left(\left(1-\nu_{1}^{2}\right)/E_{1}\right)+\left(\left(1-\nu_{2}^{2}\right)/E_{2}\right)\right)}{\left(\left(1/D_{1}\right)–\left(1/D_{2}\right)\right)}\right]}^{2/3}. \end{array}$$

However, the complex geometries, material properties, and loads of real-life joints make the Hertzian theory of limited value, while sophisticated analytical and finite element models still require experimental verification [5–7]. Thus, experimental techniques for studying joint contact mechanics remain the “gold standard.” For instance, the most widely used in vitro experimental method involves inserting a thin pressure-sensitive film into the joint space (e.g., Fujifilm or Tekscan) [8–10]; however, the film's finite thickness disturbs the joint's ordinary articulation. Similarly, the most widely used in vivo experimental method uses video recording of 3D limb motion during some prescribed activity combined with inverse dynamic analysis of a 3D link-segment model to compute joint contact force (e.g., walking gait or workplace tasks) [11–13]; however, this does not generate joint contact area or stress distribution.

Unfortunately, few review articles have been published on experimental or computational methods to study the contact mechanics of natural or artificial joints. One of these only surveyed the use of instrumented implants and mathematical models to determine in vivo knee loads [14]. Some other survey papers were concerned mainly with computational techniques for mimicking in vivo knee or hip contact mechanics with only supplemental comments on experimentation [15, 16]. Furthermore, many contemporary investigators may not even be aware of the various older and newer experimental techniques for studying both in vitro and in vivo joint contact mechanics that may be ideal for their particular research application.

Therefore, this article surveys a wide variety of in vitro and in vivo experimental methods for studying the contact mechanics of different joints. Firstly, the PubMed database was searched for English-language articles dating from 1970 onward. Secondly, article reference lists were probed for extra research articles, conference papers, book chapters, industry reports, and academic theses. Thirdly, only select resources were cited for each experimental method to eliminate redundancy and minimize the number of citations. Consequently, this paper (a) describes the basic working principles, pros, and cons of each method, (b) examines trends among studies and techniques, and (c) makes recommendations for future directions. This paper hopefully informs biomechanics investigators about various experimental methods useful for studying joint contact mechanics.

## 2. Survey of Experimental Methods

### 2.1. Types of Methods

**Figure 2:**
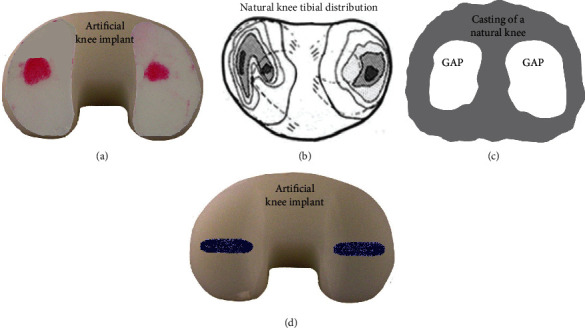
Mechanochemical methods for joint contact mechanics research: (a) Fujifilm; (b) microindentation film [17]; (c) casting material; (d) staining dye. Images are used by permission of the indicated citations; otherwise, they are original to the current authors.

**Figure 3:**
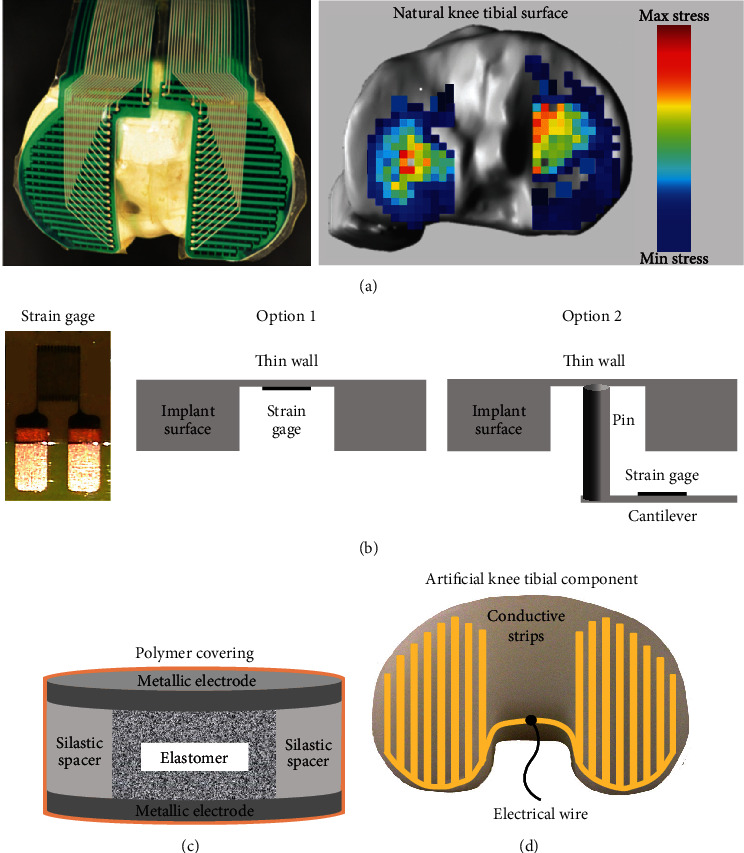
Electronic sensor methods for joint contact mechanics research: (a) Tekscan [9]; (b) strain gages; (c) piezosensors; (d) e-coating. Images are used by permission of the indicated citations; otherwise, they are original to the current authors.

**Figure 4:**
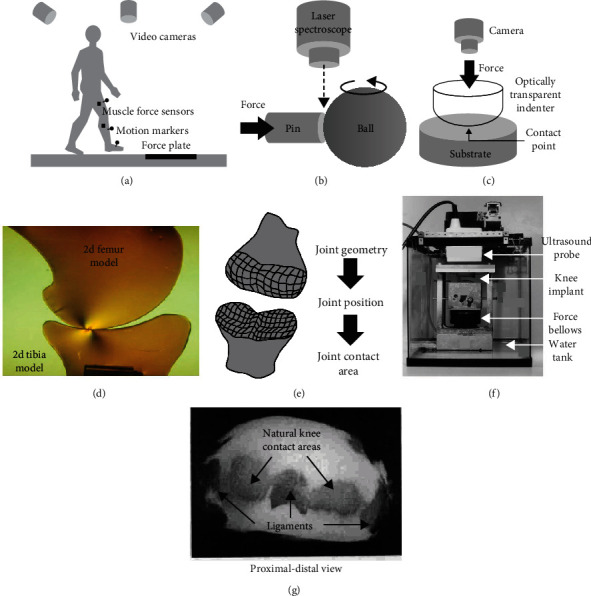
Noninvasive methods for joint contact mechanics research: (a) videography; (b) laser spectroscopy; (c) optical visualization; (d) photoelasticity [18]; (e) proximity; (f) ultrasound [19]; (g) X-rays [20]. Images are used by permission of the indicated citations; otherwise, they are original to the current authors.

Experimental techniques for investigating in vitro or in vivo joint contact mechanics have been classified into 3 categories based on their principles of operation. Mechanochemical methods use thin films or chemical substances that physically deform inside or around the joint in proportion to mechanical load (Figure 2) [17]. Electronic sensor methods use individual transducers or arrays of transducers inside or around the joint whose electrical properties change in proportion to mechanical load (Figure 3) [9]. Noninvasive methods use approaches that do not involve inserting a device or substance into the joint, thereby avoiding disruption of joint articulation (Figure 4) [18–20]. A description is given below of each technique's basic working principles, strengths, and weaknesses. Also, some important characteristics of all methodologies are summarized in tabular form for comparison (Table 1).

Several caveats should be noted. Firstly, almost all of the techniques have already been used or can potentially be applied to a variety of natural or artificial joints although the most commonly studied joints have been the shoulder, elbow, hip, and knee because they are the largest joints in the human body. Secondly, a few methodologies have only been used on idealized geometries and materials that partially mimic real-life natural or artificial joints; thus, they need to be developed further. Thirdly, numerical values for accuracy, repeatability, resolution, and financial cost are not given here, since they may either be unknown, highly dependent on the particular version of the device, and strongly related to the specific joint application and may change in the future.

### 2.2. Mechanochemical Methods

#### 2.2.1. Fujifilm

This device is made of 2 thin flexible films made of polyester, namely, a transfer film that has surface microcapsules of 2 to 26 *μ*m diameter and a developer film (Figure 2(a) and Table 1) [6, 8, 10, 19, 21–36]. This is the most commonly used mechanochemical method for studying the contact mechanics of joints. Initially, care is taken to avoid premature contact between the 2 sheets to prevent untimely chemical reactions. The films can then be individually cut using scissors or a knife to fit joint geometry, thereby avoiding sheet wrinkling during tests. The films can then be stacked together without pressing them together, thereby forming a 0.2 mm thick composite film that is inserted into the joint. Pen marks can be placed at key spots to identify the location of the film relative to the joint. When static compressive joint force is applied, the transfer film's microcapsules will burst releasing a colorless liquid that reacts with the developer film. The result is a reddish area on the developer film that represents the maximum joint contact area generated at maximum force. Photographs or flatbed scans can be taken of the developer film for analysis using appropriate computer imaging software. The software can be used to measure the contact area using either a “tracing” or “thresholding” method, as well as obtaining average contact stress and contact stress distribution. The device is also available as a single film that has a microcapsule layer immediately adjacent to a developer layer. Notably, an image of the film's 2D contact results could be “virtually” overlaid on top of a 3D computer model of the joint surface created using computed tomography (CT), magnetic resonance imaging (MRI), laser scanning, etc., to get the 3D contact area and stress distribution. Even so, the film thickness disturbs joint articulation. Also, although a fluctuating dynamic force could be used, the contact area would only be the maximum value achieved during loading, so the film is not ideal for dynamic joint testing. Finally, this method is also very easy and quick to use because it only requires minimal preparation of the sheets before insertion in joint space, but it is not reusable because the colored area formed during compression is permanent.

#### 2.2.2. Microindentation Film

This item is fabricated using 2 thin flexible films made of a Mylar diaphragm/nylon screen and an alkyd paint/acetate substrate (Figure 2(b) and Table 1) [17, 37, 38]. As with other mechanochemical films, care must be taken to prevent mechanical stressing from premature contact between the 2 microindentation sheets. Microindentation films can be easily shaped to match the joint's contours to avoid sheet wrinkling with scissors or a knife, and they are stacked on top of each other without pressing them together, thereby creating a 0.285 mm thick composite film that is placed into the joint space. The film's position relative to the joint can be determined from pen marks. As static compressive joint force is applied, the nylon screen layer presses against the alkyd paint layer to create an indentation pattern. Image analysis involves sandwiching the indented layer between glass plates, beaming a light source at an angle onto the indented layer to reveal the indentation pattern, outlining the indentation pattern by pen onto the glass plate, and then taking photographs. This indentation pattern represents the maximum joint contact area generated at maximum force, which can be further analyzed to obtain average contact stress and stress distribution but is not ideal for dynamic joint testing. The film's 2D contact results can be superimposed onto a 3D computer model of the joint surface to obtain 3D contact area and stress distribution. However, the film's thickness disrupts natural joint articulation, and unlike Fujifilm, the microindentation film method was designed and built for specific knee joint contact mechanics studies and, therefore, is not commercially available.

#### 2.2.3. Casting Material

This is a thick sludge or flowing liquid, such as medical cement, industrial cement, silicone rubber, or similar mouldable substance (Figure 2(c) and Table 1) [21, 39–42]. A thicker casting material can be hand-packed onto the separated and exposed joint surfaces. However, a flowing liquid must be poured into a chamber that surrounds the separated and exposed joint surfaces. A static compressive joint force is then applied to squeeze out excess casting material away from the actual contact interface. The casting material is allowed to dry or set according to the material's manufacturer instructions. The joint surfaces are then separated, so the final casting can be removed. The result is a space or void in the casting that represents the maximum joint contact area that was generated at maximum force. This contact area can be traced by pen onto a sheet of paper, scanned using a flatbed scanner, or photographed for further analysis using optical methods or computer imaging software, but this gives a 2D result of a 3D quantity. Alternatively, a 3D laser scan image could be obtained of the actual joint surface with the casting in place for analysis using computer imaging software to obtain the 3D contact area. However, there is a disturbance of joint articulation until the casting material is fully squeezed out allowing for contact stress averages to be found but not the contact stress distribution. Lastly, casting is not an ideal method for dynamic joint testing as the contact area would only be the maximum value achieved during loading.

#### 2.2.4. Staining Dye

This liquid could be artist's ink, machinist's blue dye, methylene blue, silicone oil plus carbon black powder, or any similar substance (Figure 2(d) and Table 1). The first option is to stain the joint's noncontacting surface, as follows [43, 44]. The joint is enclosed in a bag or container. A static compressive joint force is then applied. The dye is poured, or infused via tubes, into the bag or container to allow rapid staining. The bag or container is then drained. The result is an unstained region that represents joint contact area. The second option is also to stain the joint's noncontacting surface, as follows [45]. The joint surfaces are coated with silicone oil plus carbon black powder. During compressive joint load, the substance squeezes away from the joint's contact area. The result is an unstained region that represents joint contact area. The third option, in contrast, is to stain the joint's contacting surface, as follows [22, 46]. The dye is applied to one joint surface. A static compressive joint force is applied, so some dye transfers from one surface to the other at the interface. The result is a stained region that represents joint contact area. For all 3 options, a grid of mesh gauze or a transparent plastic sheet can be placed over the joint surface, the contact area is traced with a pen, a photograph or flatbed scan is taken of the gauze or sheet, and computer imaging software is used for analysis giving a 2D result for a 3D quantity. By using computer imaging software, a 3D laser scan image could be obtained of the actual joint surface's 3D contact area for analysis. Yet, there is a temporary disturbance of joint articulation while the staining dye is being squeezed. Also, staining is not an ideal method for dynamic testing as the contact area would be the minimum (i.e., first option) or the maximum (i.e., second and third options) achieved during loading. Finally, this method requires minimal preparation with widely available materials, but the condition of the biological tissue impacts the quality of the staining on natural joint surfaces.

### 2.3. Electronic Sensor Methods

#### 2.3.1. Tekscan

This device is a 0.1 to 0.2 mm thick polyester sheet composed of a grid of mutually orthogonal strips made from electrically conductive silver ink (Figure 3(a) and Table 1), and this is the most frequently used electronic sensor used for studying the contact mechanics of joints [9, 10, 25, 36, 47–52]. The sheet can be easily cut so that its geometry matches the intended joint, thereby avoiding sheet wrinkling during loading. The sheet is then inserted into the joint space. Measurements can be made at key spots to identify the exact location of the film relative to the joint. Upon application of static or dynamic compressive joint force, the electrical resistance of the strips changes in proportion to the magnitude of the force. The changes in the electrical signal are sent through wiring to the appropriate electronic setup. Dedicated computer software is then used to process and convert electrical information into graphic form. The result is a 2D matrix of colored pixels, or a 3D diagram if overlaid on top of a 3D computer model, representing the joint's contact area, average contact stress, and contact stress distribution, as well as the potential to compute the weighted center of pressure. Nonetheless, the film thickness disturbs joint articulation and may not provide enough resolution for some applications due to pixel size, which is related to grid density. Finally, they may be reused with proper care and if the forces applied are minimal, but Tekscan requires dedicated software making it costlier and more challenging to use than some mechanochemical methods.

#### 2.3.2. Strain Gages

This device most commonly employs metal foils or wires arranged in a grid pattern, whose electrical resistance changes under mechanical strain (Figure 3(b) and Table 1). Strain gages can be mounted in custom-made hip implants [53–55] or knee implants [56–58]. There are several possible ways to do this. The first option is to partially mill one or several holes into the implant to create one or several thin walls at the articulating surface so that a strain gage can be attached to the nonarticulating underside of each thin wall. The second option is to insert a metal pin that contacts each thin wall and a cantilever beam equipped with a strain gage. The third option, in contrast, is to place strain gages at strategic locations away from the articulating surface, like the hollow neck of a hip stem or underneath the metal tibial tray in a knee implant. Then, static or dynamic joint loading will proportionally change the strain gage's electrical resistance. Each strain gage's electrical signals are then sent by wires or wireless radio telemetry to electronic instrumentation and/or computer software for analysis. Depending on the number, location, and arrangement of strain gages, the final result may be a single contact force, multiple contact forces, or potentially an estimate of contact area, average stress, and stress distribution. Moreover, one joint surface must be artificial to accommodate strain gages, so the technique is not applicable when both joint surfaces are natural. Artificial joints equipped with strain gages are commercially available for some joints, but not for all anatomical locations. Additionally, strain gages have been used in vivo subjects; however, they require expertise and high precision to ensure accurate and consistent measurements.

#### 2.3.3. Piezo sensors

Piezoelectric and piezoresistive sensors are made of semiconductor material (e.g., barium titanate, germanium, quartz, and silicon) sandwiched between metallic electrodes so that a mechanical strain causes, respectively, a generation of electrical voltage or a change in electrical resistance (Figure 3(c) and Table 1). These sensors have been used in a variety of ways for measuring contact stresses for natural and artificial joints [59–62], as well as internal spine disc pressures during intervertebral articulation [63–65]. Specifically, the sensors can potentially be mounted into cartilage, bone, or artificial materials via predrilled holes, or they can be directly inserted by a needle or probe into or under the joint cartilage or spinal disc. Then, when static or dynamic loading is applied during joint or intervertebral articulation, the sensor properties are altered. The electrical signals are simultaneously sent to the appropriate electronic instrumentation for conversion to force or stress values. The use of a single sensor limits this stress value to one single location, whereas multiple sensors at key locations could allow for an estimation of contact area and stress distribution over a larger region. Even so, this would still only provide a 2D result that represents a 3D quantity. Furthermore, joint articulation can be somewhat disrupted if the sensors are placed too close to the top surface of the joint. With piezo sensors, measurements can be recorded in real time enabling dynamic loads to be assessed throughout an entire test, but they take time and expertise to use effectively.

#### 2.3.4. e-Coating

This technique uses an electrically conductive coating to measure the contact area of an artificial knee joint (Figure 3(d) and Table 1) [35, 66]. In particular, a sputter coater is used to apply a gold/palladium coating that is 400 angstroms thick onto the articulating surface of a knee replacement's polymer tibial component. This coating could be arranged in strips that are 1 mm wide or cover a single larger region on each tibial condyle. A wire is then attached to the polymer tibial component at the intercondylar eminence, the metal femoral component at the intercondylar notch, and appropriate electronic instrumentation. Static compressive joint force is applied to articulate the femoral and tibial components, while simultaneously recording changes in electrical resistance between the components. The electrical resistance value is finally converted to a contact area size based on a calibration curve from prior compression tests on flat polymer plates with known contact areas and contact stress. However, it is not possible to identify the shape of the contact area or its location relative to the joint replacement. Also, joint articulation is somewhat disrupted by the presence of the e-coating on the joint surface. Moreover, a fluctuating dynamic force could potentially be applied causing the electrical resistance and, hence, the contact area size to fluctuate dynamically in real time, although this was not evaluated in the cited studies.

### 2.4. Noninvasive Methods

#### 2.4.1. Videography

This well-established technique combines video recording of human limb movements, electromyography for muscle force measurements, and mathematical computations to estimate natural or artificial joint contact forces (Figure 4(a) and Table 1) [11–13]. This is the most widely used noninvasive method for studying the contact mechanics of joints. In particular, passive reflective or active infrared markers are placed at various locations on the limbs (e.g., arm or leg) of a live volunteer, while electromyography sensors are placed on the skin next to the muscles or inserted directly into the muscles. The volunteer is then asked to perform a common action, such as a daily activity (e.g., walk across a walkway, sit/rise from a chair, and ascend/descend a staircase), a sports movement (e.g., run across a runway, jump on a platform, and kick a ball), or a workplace task (e.g., push a door, pull a handle, and lift a box). During these actions, one or several high-speed or infrared video cameras record the 2D or 3D motions of the markers to provide linear and angular positions, velocities, and accelerations of the limb. Moreover, electromyography sensors monitor the electrical intensity and timing of muscle activity. Also, load sensors on the physical objects with which the volunteer directly interacts (i.e., walkway, platform, and handle) provide load data. Then, a step-by-step mathematical process called “inverse dynamic analysis” uses limb motion data and physical object loads as inputs for equilibrium equations to calculate joint “external” forces and moments. Next, these “external” forces and moments, along with muscle forces, are also inputted into equilibrium equations to solve for joint “internal” contact forces in normal and shear directions. However, the process involves multiple assumptions, devices, and stages each with errors that can cause a much larger compounded error in the final result. Also, contact area or contact stress data cannot be obtained, in vitro specimens cannot be measured, and it requires specialized equipment with high financial costs.

#### 2.4.2. Laser Spectroscopy

This approach uses a laser-based optical microscope to monitor a known contact point at the interface of an artificial hip joint (Figure 4(b) and Table 1) [67]. Specifically, the femoral ball of an artificial hip joint is mounted into a specially designed pin-on-ball wear tester. The pin of known geometry represents the acetabular liner material, while the ball of known geometry represents the femoral ball material. A known axial force is then applied to the pin to push it against the femoral ball, while the femoral ball rotates continuously in a tangential direction to the pin in order to simulate hip joint sliding wear. Simultaneously, a laser-based optical microscope sends a laser directly at the pin-on-ball contact point and then monitors the amount of laser scattering. The information is analyzed using a computer software algorithm to obtain the compressive stress at the contact point vs. sliding distance (i.e., time). In particular, it should be noted that the contact stress results are affected by the heat generated by joint wear during sliding; thus, this must be taken into account via a calibration factor. Also, pin-on-ball contact may not represent the actual shape of the contact area for a natural or artificial joint but is mainly used for joint wear simulation, while contact stress distribution cannot be experimentally obtained.

#### 2.4.3. Optical Visualization

This methodology allows direct visualization of the contact area of a partially transparent artificial joint (Figure 4(c) and Table 1). One study [68] fabricated an optically transparent “femoral” indenter from a plano-convex spherical lens and a glass block, along with an “acetabular” silicone rubber layer. Static compressive force was applied at different strain rates to press the surfaces together. Videos and photographs of the contact area were obtained using a mirror placed 45° above the indenter and then measured using a profile projector. Contact areas were very comparable to Hertzian theory over a range of subclinical and clinical-level loads. However, the elastic modulus of many glasses (50 to 130 GPa) [69] overlaps with metals used for a hip replacement's femoral component (120 to 230 GPa) [70], while the elastic modulus of the silicone rubber (4 MPa) that was used overlaps with a natural joint's cartilage (1.6 to 220 MPa) [70]. Thus, the study somewhat simulated a hip hemiarthroplasty or a hip cushion-type bearing implant. Another group [71, 72] used a transparent polyester resin to make a geometrically realistic “tibial” insert of a knee replacement, whereas the “femoral” condyles were made of 2 steel spheres. However, the elastic modulus of the “tibial” insert material (550 MPa) was far lower than the ultra-high-molecular weight polyethylene used in actual knee replacements (1240 MPa) [70]. Static and dynamic compressive forces were applied, a lubricating ink improved image contrast, a light source illuminated the articulation, and a camera recorded images. Contact areas were similar to Hertzian theory over a range of subclinical and clinical-level loads. Even so, the technique has not yet been used with geometries and/or materials that fully represent natural or artificial joints. Also, the results from this technique are 2D values representing 3D quantities.

#### 2.4.4. Photoelasticity

This technique uses test objects made from noncrystalline, transparent, optically isotropic materials that become optically anisotropic during mechanical stress, so the resulting stress “fringe” pattern can be seen using a polariscope (Figure 4(d) and Table 1). One study did 2D experiments replicating a knee joint [18]. Columbia Resin 39 (i.e., CR-39) was used to make a synthetic femur and tibia with an elastic modulus of 2.1 GPa and ultimate tensile strength of 21 MPa, which overlaps with human cancellous bone [70]. The synthetic femur and tibia were 2.5x larger than human bones, but they were 2D specimens of finite thickness. Static compressive knee loads (0.5 to 3 kg) and flexion angles (0° and 30°) produced concentric stress lines emanating from knee contact points. Also, static compressive knee loads (30 and 80 kg) and flexion angles (0° and 30°) generated contact stress distributions similar to linear and nonlinear analytical models. Another study did 3D experiments mimicking a hip joint [73]. The investigators made a synthetic femur using epoxy resin, applied static compressive hip joint load in an oven at an elevated temperature, allowed the specimen to cool so its stress state would be maintained (i.e., “stress freezing”), and then physically cut the specimen into slices to image the stress pattern at several planes. Results showed concentric stress lines emanating from 2 contact points on the femoral head where the load was applied. Nonetheless, photoelasticity requires test objects to be made from only certain synthetic materials; therefore, it cannot be used for natural joints. Although 2D photoelastic tests can potentially be done using dynamic loads, 3D experiments require using the “stress freezing” strategy under static loads.

#### 2.4.5. Proximity

This technique obtains accurate joint surface geometries, monitors the mutual proximity of joint surfaces during in vitro tests [39, 74–76] or in vivo tests [58, 77–79], and then calculates joint contact areas (Figure 4(e) and Table 1). Before or after testing, 3D models for the geometries of natural or artificial joints are obtained from CT, MRI, X-rays, laser scans, or photographs (i.e., stereograms or stereophotograms). Then, for in vitro tests, natural or artificial joints are mounted in a 2D or 3D joint loading machine, while the joint loading machine's sensors, magnetic tracking, photographs, or videos are used to monitor the position of the joint during articulation. Alternatively, for in vivo tests, live subjects with natural or artificial joints are asked to perform specific joint movements, while a fluoroscope emits X-ray pulses to capture multiple images per second of the joint movement from multiple anatomical directions. Then, a computational algorithm combines the known geometries and positions of the joint in order to calculate the size, shape, and/or location of the “intersecting” 3D contact area of the articulating joint. Next, if relevant and possible, the joint's material properties can be incorporated into the computational algorithm to calculate joint contact force and/or stress distribution. However, the process employs multiple assumptions, devices, and stages each with errors that can create a much larger compounded error in the final result.

#### 2.4.6. Ultrasound

This method employs sending and receiving sound waves to measure the contact areas of natural or artificial joints (Figure 4(f) and Table 1). One approach uses diagnostic ultrasound imaging on artificial knee joints [19, 80]. Specifically, a test jig with a loading mechanism and ultrasound viewing window is placed into a chamber. The knee joint's metal femoral component and polymer tibial component are then mounted into the jig. The chamber is then filled with water for better ultrasonic transmission; but ongoing infusion of ultrasound gel onto the ultrasound probe and joint surfaces could work. Static compressive joint force is then applied, after which a linear ultrasound probe is moved in increments across the viewing window to capture images. Ultrasound images are then analyzed using computer software to reconstruct a 2D contact area and its location relative to the joint, or potentially even a 3D contact area if correction factors are applied. An alternative approach utilizes an individual ultrasonic transducer mounted inside a specially designed metal femoral head [81, 82]. Fluctuating dynamic hip joint loading is then applied using a joint simulator. The ultrasonic transducer detects thickness changes in the natural acetabulum's cartilage or the artificial acetabulum's polymer liner, but it could easily be applied to monitor the amount of interfacial contact. Even so, an average contact stress could be calculated from the ultrasound contact area but not the contact stress distribution nor provide adequate resolution for certain applications.

#### 2.4.7. X-Rays

This technique employs X-rays that are usually used for clinical diagnosis in order to image the contact zone of a joint (Figure 4(g) and Table 1). The goal is to use this as a stand-alone method, rather than combining it with computational models, instrumented joint replacements, or thin films, as described earlier. An older classic approach involves injecting the joint of a cadaveric specimen or a live subject with barium sulfate solution to act as a contrasting medium [20, 83]. While the subject or specimen remains in a static position, X-rays are taken of the joint's articulating surface from different anatomical directions. The radiolucent region of the X-ray image contains no barium, thereby representing the joint's contacting surfaces. Measurements can then be made from the X-ray images manually to reconstruct a 2D or 3D contact area, as well as the location relative to the joint. However, a more modern version of this approach could potentially involve utilizing high-speed X-rays of the subject's joint without injecting a contrasting agent, while they are engaged in a dynamic physical activity [84, 85]. The X-ray images could then be further analyzed using appropriate computer imaging software to reconstruct a 2D or 3D contact area, along with its location with respect to the joint. Similar steps could be applied during in vitro tests on cadaveric joints with or without joint replacements. Also, CT or MRI scanning can potentially be used in a similar way. Even so, it should be noted that X-ray images show an overlap at the interface between the 2 articulating surfaces of the joint, but the resolution limits of the X-ray image may not permit identifying the exact contact area. In addition, average contact stress cannot be obtained for in vivo tests since joint force is unknown, but stresses can be acquired for in vitro tests since a known joint force is used.

## 3. Discussion

### 3.1. General Observations

Researchers could choose a suitable experimental method for their joint contact mechanics application by directly comparing each technique's characteristics (Table 1). Some characteristics are more qualitative since the technique either does or does not have a particular capability. This includes output data (i.e., does it provide contact force, area, and/or stress?), data type (i.e., can it give static and/or dynamic results?), specimen type (i.e., can it be used in vitro and/or in vivo?), reusability (i.e., is it multiple or only single use?), disruption of articulation (i.e., does it interfere with joint articulation?), simplicity (i.e., is it easy to use?), and commercial availability (i.e., can the device can be purchased or must the device be tailor-made by the researcher?). In contrast, other characteristics are more quantitative, since they could be assigned a numerical value. This includes accuracy (i.e., what is the percentage error compared to a known value?), repeatability (i.e., what is the percentage variation from test to test?), resolution (i.e., what is the smallest measurable value?), and financial cost (i.e., what is the purchase price?). Newer investigators may wish to consider a potential experimental method that is more versatile due to its larger number of capabilities; but more experienced researchers may feel that the experimental method they already use is perfectly suitable. This review is only a preliminary attempt to compare these methodologies to each other; a proper comparison would include performing joint contact mechanics tests under the same conditions using as many techniques as possible.

There were many different testing conditions employed by joint contact mechanics studies. Specifically, there were a variety of measurement methods (e.g., Fujifilm or piezoelectric sensors), joint types (e.g., natural or artificial), joint qualities (e.g., normal or osteoarthritic), joint locations (e.g., shoulder or knee), joint angles (e.g., flexion or extension), specimen types (e.g., cadaveric specimen or living subject), load types (e.g., static or dynamic), load magnitudes (e.g., subclinical or clinical), activity types (e.g., level walking or stair climbing), etc. Clearly, this illustrates the abundant opportunities and strategies available to researchers for joint contact mechanics research. Conversely, this large variety also makes it extremely difficult for investigators to quantitatively or even qualitatively compare measurements to prior published findings in order to verify particular data, add new scientific knowledge, create an overarching theory, etc.

The diverse goals researchers have will influence the technique researchers choose to study joint contact mechanics. In Table 1, (i) in vitro studies on artificial joints are typically concerned with mechanical stresses and wear generated on the joint surface (e.g., Fujifilm [8], Tekscan [27], and strain gages [55]), (ii) in vitro studies on biological joints are commonly interested in understanding the basic functionality of the joint as a baseline for other research (e.g., casting material [18], Tekscan [9], and piezo sensors [62]), and (iii) in vivo studies on artificial or biological joints may be focused on the normal and shear loads (i.e., kinetic) and the linear/angular motions (i.e., kinematics) of joints during normal daily activities of human volunteers and/or the progression and influence of joint diseases like osteoarthritis (e.g., videography [11], proximity [60], and X-rays [84]). Similarly, the parameter that is of interest to the research will depend on the research question. For instance, average and peak contact stress for in vitro studies is critical for understanding the risk of failure or wear of artificial joint surfaces if their material strength limits are exceeded [8], whereas a correct contact area (location) may be important for in vivo studies to ensure that there is no loosening of the artificial joint surfaces in the patient [78].

The vast majority of joint contact mechanics investigations only used one experimental methodology to conduct their research. In some cases, investigators performed their own experimental calibration tests using known values for joint contact force, area, or stress, and/or they compared their data to very similar prior publications to give credibility to their findings. Unfortunately, some investigations simply assumed that their methodology was reliable because of previous verification done by other researchers and/or the technical specifications provided by the technology's manufacturer. Even so, such validations are not always critically important for studies only concerned with the relative differences in joint contact force, area, or stress between test groups (e.g., implant 1 vs. 2 vs. 3), rather than the absolute reliability of magnitudes.

There were a few methodologies that have only been used on idealized geometries and materials that only partially mimic real-world natural or artificial joints. Similarly, some of the protocols utilized special test setups that were manufactured by the researchers themselves, since some of the components were not commercially available. This includes e-coating, laser spectroscopy, optical visualization, photoelasticity, and ultrasound. But this was understandable, since the goal of those studies was mainly to provide a “proof of principle” for the basic concept of their novel methodology. Of course, those techniques would need to be developed further to be useful for joint contact mechanics studies by the broader research community. Nevertheless, these methods were included in this review because they are potentially beneficial tools.

Although the scientific and technological aspects of joint contact mechanics research are critical, some concepts regarding the ethical use of the reviewed methods should be highlighted. For instance, nine methods can only be practically used in vitro artificial or biological joints such as Fujifilm, Tekscan, and photoelasticity (Table 1). Moreover, if they were attempted on live subjects, this could cause physical harm, and thus, they should not be considered ethically proper to use. In contrast, the other 6 methods have been commonly used on live subjects to study joint contact mechanics, such as videography, strain gages, and X-rays (Table 1). Even so, researchers need to obtain permission from their institutions' ethics research committee to ensure that study protocols meet the highest standard for medical ethics. These standards should not be considered as limitations on the researcher's ability to conduct their investigation, but rather medical ethics standards should be seen as providing a proper scope for inquiry as done in Good Clinical Practice (GCP) or the Declaration of Helsinki principles.

### 3.2. Future Directions

More studies are needed that directly compare multiple experimental techniques with each other or against theoretical data in order to better understand their relative performance for joint contact mechanics research. To date, most of these comparative investigations have been focused on Fujifilm or Tekscan. For instance, Fujifilm has been compared to Tekscan [10, 25, 31, 36], castings [21, 22, 39], dyes [22, 39], e-coating [35], proximity [39], ultrasound [19], Hertzian theory [31], and finite element models [28, 29, 31, 33]. Similarly, several studies compared Tekscan vs. a mechanical tester's load cell [36], a Hertzian solution [7], and a finite element model [7], as well as a Tekscan system with lower vs. higher resolution [48]. Additionally, comparisons have also been done for proximity vs. castings and dyes [39], microindentation film vs. Hertzian theory [37], an optical method vs. Hertzian theory [68, 71, 72], and photoelasticity vs. analytic models [18]. In contrast, the vast majority of studies used only one experimental technique without comparison against other experimental or theoretical approaches, since they were mainly focused on a particular joint contact mechanics topic, rather than comparing methodologies.

Technologies presently used for other biomechanics applications could be further developed for joint contact mechanics research. This would give investigators additional experimental tools. For example, thermographic stress analysis (TSA) [86, 87] and digital image correlation (DIC) [88, 89] have been successfully used for full-field stress mapping of whole bones, soft tissues, and orthopaedic implants. The pros are that they can obtain high-resolution stress distributions across the joint, their noninvasiveness avoids disrupting joint articulation, and they are commercially available. The cons are that they require a direct “line of sight” to the joint surface, they would be limited to in vitro joint tests, and they may be too financially costly for some budgets. Even so, TSA and DIC imaging could potentially be done on an exposed joint surface before loading (i.e., baseline) and after loading (i.e., test case) to prevent substantial thermal cooling (i.e., to avoid TSA errors) or stress relaxation (i.e., to avoid DIC errors) of the joint surface.

Similarly, fiber optic sensors change how they reflect ultraviolet light along their length when they undergo tensile or compressive strain [90, 91]. They have been used at various interfaces to measure axial load at one location within a porcine knee meniscus [92], spine disc internal pressure [93], and bone-implant interface stress [94]. Their main strengths are small size, high precision, biocompatibility, no damage done to any electronic components even under high strain, and no interference from electromagnetic sources. Their main weaknesses are sensitivity to temperature change and different responses to tensile and compressive strain. Even so, multiple individual fiber optic sensors, or in the form of a rosette pattern, could potentially be inserted into the joint space or in adjacent layers to measure contact loads at several locations.

Internationally recognized standards could be established for using some of the above experimental techniques specifically for joint contact mechanics research. At present, investigators usually rely on previously published journal articles, book chapters, conference papers, industry reports, manufacturer instructions, academic theses, and their own subjective experience on how to best utilize these methodologies. As such, some time, effort, and resources could be given to developing standardized protocols for or by organizations like the American Society for Testing and Materials (ASTM) [95] and the International Organization for Standardization (ISO) [96]. Of course, such protocols will need to be occasionally updated in order to keep up with advancements in that particular technology [97]. Even so, standardized protocols could help bring some cohesion to the large variety of testing regimes used to date in order to improve the quality of joint contact mechanics studies. Moreover, widely accepted procedures for these techniques could also help researchers reliably compare their findings with other reports to build a consensus view or larger theory on certain topics for joint contact mechanics.

### 3.3. Potential Limitations

This review article had possible weaknesses. Only English-language literature was surveyed, so potentially useful findings were missed. Experimental methodology was the central topic, although some remarks were provided regarding the pros and cons of analytical and finite element models [7]. Joints like the shoulder, elbow, hip, and knee were the primary concern, but the techniques could potentially be used for other biomechanical interfaces like tooth-on-tooth biting, bone-implant interface, and prosthetic limb-stump interface. Quantifying the basic contact forces, areas, and stresses of artificial joints was one primary theme, whereas wear debris measurement is a separate topic that may involve internationally recognized testing protocols for entire artificial joints (i.e., 6-degree-of-freedom joint simulator) or artificial joint materials (i.e., pin-on-disc setup) [98]. To avoid giving misleading information, numerical values were not stated here for accuracy, repeatability, resolution, and/or financial cost of the experimental techniques; even so, the reason is that such quantities are either unknown, vary widely based on the specific version of the device, are highly dependent on the particular joint application, and can easily change in the future. Finally, although some of the reviewed technologies could be considered outdated, the goal was to inform readers of older and newer technologies, so they can decide what may be the most useful methodology for their particular joint contact mechanics research.

## 4. Conclusions

This article surveyed over 50 years of English-language literature on in vitro and in vivo experimental techniques used to measure the static or dynamic contact forces, areas, and stresses at the interface of natural and artificial joints, like the shoulder, hip, and knee. It described the basic working principles, strengths, and weaknesses of each methodology. It examined the general trends found among the investigations and techniques. It also offered suggestions about future work topics and developing even newer methodologies. This review paper will hopefully inform biomechanics researchers about the various available experimental methodologies for studying joint contact mechanics.

## Figures and Tables

**Table 1:** Characteristics of experimental techniques for joint contact mechanics research.

Characteristic	Contact force	Contact area (size)	Contact area (location)	Contact stress (average)	Contact stress (distribution)	Static data	Dynamic data	In vitro specimens	In vivo subjects (ethical use)	Reusable for multiple tests	Joint contact is undisturbed	Easy and quick to use	Commercially available	Financial cost to purchase	Accuracy (i.e., error)	Repeatability (i.e., variation)	Resolution (i.e. smallest result)
*Mechanochemical methods*																	
Fujifilm	N	Y	Y	Y	Y	Y	N	Y	N	N	N	Y	Y	L	V	V	V
Microfilm	N	Y	Y	Y	Y	Y	N	Y	N	N	N	Y	N	L	V	V	V
Casting	N	Y	Y	Y	N	Y	N	Y	N	N	T	Y	Y	L	V	V	V
Staining dye	N	Y	Y	Y	N	Y	N	Y	N	N	T	Y	Y	L	V	V	V

*Electronic sensor methods*																	
Tekscan	N	Y	Y	Y	Y	Y	Y	Y	N	Y	N	N	Y	M	V	V	V
Strain gages	Y	Y	Y	Y	Y	Y	Y	Y	Y	Y	Y	N	Y	M	V	V	V
Piezosensors	Y	Y	Y	Y	Y	Y	Y	Y	Y	Y	N	N	Y	M	V	V	V
e-Coating	N	Y	N	Y	N	Y	Y	Y	N	Y	N	N	P	M	V	V	V

*Noninvasive methods*																	
Videography	Y	N	N	N	N	N	Y	N	Y	Y	Y	N	Y	H	V	V	V
Laser spectro.	N	N	N	Y	N	N	Y	Y	N	Y	Y	N	P	M	V	V	V
Optical	N	Y	Y	Y	N	Y	Y	Y	N	Y	Y	N	P	M	V	V	V
Photoelasticity	N	Y	Y	Y	Y	Y	Y	Y	N	Y	Y	N	P	M	V	V	V
Proximity	Y	Y	Y	Y	Y	Y	Y	Y	Y	Y	Y	N	Y	H	V	V	V
Ultrasound	N	Y	Y	Y	N	Y	Y	Y	N	Y	Y	N	P	H	V	V	V
X-rays	N	Y	Y	Y	N	Y	Y	Y	Y	Y	Y	N	Y	H	V	V	V

Key: Y: yes; N: no; T: temporary joint contact disturbance by the device during loading to maximum force; P: parts of the equipment or setup are commercially available, but some of it has to be custom-made by the researcher; L: low financial cost; M: medium financial cost; H: high financial cost; V: variable or unknown quantities that are dependent on the particular version of the device or testing protocol, so magnitudes are not provided here to avoid giving misleading information.
